# Clinical characteristics and prognosis of familial non-medullary thyroid carcinoma: a retrospective study of 98 families

**DOI:** 10.1186/s12957-026-04357-9

**Published:** 2026-04-15

**Authors:** Guang Yang, Yujie Zhang, Tianfeng Xu, Yuanyuan Fan, Yanhao Ran, Pengyu Li, Xun Zheng, Tao Wei

**Affiliations:** 1 Division of Thyroid Surgery, Department of General Surgery, West China Hospital, Sichuan University, Chengdu, 610041 China; 2 Department of Medical Ultrasound, West China Hospital, Sichuan University, Chengdu, 610041 China

**Keywords:** Familial, Sporadic, Non-medullary, Thyroid carcinoma, Clinicopathologic features, Prognosis

## Abstract

**Background:**

The differences in clinicopathological features and prognosis between familial non-medullary thyroid carcinoma (FNMTC) and sporadic non-medullary thyroid carcinoma (SNMTC) remain a subject of debate. This study aims to compare the clinicopathological features and recurrence-free survival (RFS) between FNMTC and SNMTC, with a particular focus on the subgroup differences based on the number of affected relatives.

**Methods:**

A retrospective analysis was conducted on 672 NMTC patients who underwent thyroidectomy at West China Hospital between 2015 and 2024. The cohort included 224 FNMTC patients (from 98 families) and 448 SNMTC patients. FNMTC patients were further stratified into families with two affected members (FNMTC-2, *n* = 160) and families with ≥ three affected members (FNMTC-3, *n* = 64) subgroups. Clinicopathological characteristics and RFS were compared among the groups.

**Results:**

Compared to the SNMTC group, the FNMTC group demonstrated a smaller tumor diameter (1.03 ± 0.60 cm vs. 1.15 ± 0.96 cm, *p* = 0.0255) but higher rates of multifocality (43.75% vs. 34.82%, *p* = 0.024), bilaterality (32.59% vs. 23.88%, *p* = 0.0163), and lymph node metastasis (52.68% vs. 44.42%, *p* = 0.0432). Subgroup analysis revealed that the FNMTC-3 group, compared to SNMTC, was associated with a younger age at diagnosis (43.76 ± 14.53 vs. 47.01 ± 11.28 years, *p* = 0.039), a smaller tumor diameter (0.89 ± 0.46 cm vs. 1.15 ± 0.96 cm, *p* = 0.0343), and increased rates of multifocality (50.00% vs. 34.82%, *p* = 0.0185), bilaterality (37.50% vs. 23.88%, *p* = 0.0195), lymph node metastasis (57.81% vs. 44.42%, *p* = 0.0444), and thyroid follicular nodular disease (57.81% vs. 44.20%, *p* = 0.0409). Furthermore, the FNMTC-3 group had a significantly smaller tumor diameter than the FNMTC-2 group (0.89 ± 0.46 cm vs. 1.08 ± 0.64 cm, *p* = 0.0343). However, no statistically significant differences in RFS were observed among the SNMTC and FNMTC groups during follow-up.

**Conclusion:**

FNMTC-3 exhibits more aggressive clinicopathological features than SNMTC, including earlier onset, higher multifocality, bilaterality, and metastatic potential. These findings support that the number of affected family members is a significant indicator of tumor aggressiveness in FNMTC. Although no difference in RFS was observed, the distinct pathological profile of FNMTC-3 warrants more vigilant surveillance.

**Supplementary Information:**

The online version contains supplementary material available at 10.1186/s12957-026-04357-9.

## Background

The incidence of thyroid cancer has been increasing annually, with an estimated 821,000 new cases and 44,000 deaths worldwide in 2022 [1]. It has now become the most common endocrine malignancy and the seventh most common cancer globally. Non-medullary thyroid carcinoma (NMTC) accounts for over 90% of all thyroid cancers [2], including papillary thyroid carcinoma (PTC), follicular thyroid carcinoma (FTC), anaplastic thyroid carcinoma (ATC), and oncocytic thyroid carcinoma (OTC).

Based on family history, NMTC can be classified into sporadic non-medullary thyroid carcinoma (SNMTC) and familial non-medullary thyroid carcinoma (FNMTC). FNMTC is traditionally defined as the occurrence of NMTC in two or more first-degree relatives within a family, without other known predisposing factors like a history of head and neck irradiation [3]. According to the recent WHO Classification of Endocrine Tumors, non-syndromic FNMTC is further specified as either ≥ 3 first-degree relatives with follicular cell-derived thyroid carcinoma or ≥ 2 first-degree relatives with PTC, with additional supportive features including early onset (age < 33 years), affected relatives aged < 45 years, multifocal/bilateral PTC, PTC in young male patients or combining with benign lesions [4].

Although FNMTC accounts for only 5%-10% of all cases [5], it is proposed as a potentially distinct clinical entity, with some studies suggesting it may harbor unique clinicopathological and prognostic features compared to SNMTC [6–10]. However, some clinical research reports no significant differences in aggressiveness or prognosis between FNMTC and SNMTC [11, 12]. Similarly, whether the risk of aggressiveness or poor prognosis increases with the number of affected individuals within FNMTC patients also remains debatable [13]. Confronting these contradictory conclusions, Charkes et al. [14] found that when a kindred has exactly two affected first-degree relatives with NMTC, the probability of it being a true familial disease is only 31–38%. In contrast, this probability rises to over 95% in kindreds with three or more affected members. Unfortunately, no definitive genetic markers are available to distinguish familial from sporadic NMTC reliably [15].

To clarify whether FNMTC and SNMTC have distinct clinical features and prognostic outcomes, this study retrospectively collected and compared clinical data and prognostic information from patients with SNMTC and FNMTC. Additionally, we further compared the clinical characteristics of patients from FNMTC kindreds with three or more affected members to those from kindreds with only two affected members. This aims to determine the impact of the number of affected relatives within a family on the aggressiveness and prognosis of FNMTC.

## Methods

### Patient population

A retrospective analysis was performed on patients diagnosed with non-medullary thyroid carcinoma who underwent thyroidectomy at West China Hospital of Sichuan University between 2015 and 2024. The NMTC diagnosis was confirmed through pathology after surgery. FNMTC was defined as having two or more affected first-degree relatives in a family, excluding cases with familial syndromes or a history of radiation exposure [3]. Information on family history was collected through patients’ self-reports at the initial hospital admission. It was identified through the hospital’s database and confirmed via inpatient records and telephone follow-ups. The study included 224 FNMTC patients from 98 families, divided into two subgroups based on the number of affected members: 160 patients (78.2%) with two affected members (FNMTC-2) and 64 patients (21.8%) with three or more affected members (FNMTC-3). For controls, 448 SNMTC patients were randomly selected from 9065 eligible patients during the same period using simple random sampling by computer-generated random numbers at a 1:2 ratio (FNMTC: SNMTC), without matching. All participants have written informed consent before treatment, and the Clinical Trial and Biomedical Ethics Committee of West China Hospital approved the study protocol.

### Study protocol

All patients underwent comprehensive medical history documentation, physical examination, and thyroid function testing. Ultrasonography (US) was initially performed to evaluate thyroid tumor features and cervical lymph node status. Ultrasound-guided fine-needle aspiration (FNA) was subsequently utilized for cytopathological confirmation of thyroid lesions or metastatic lymph nodes. We also performed CT and radioiodine scans to assess pulmonary metastases and mediastinal lymph nodes when necessary. The surgical recommendations for patients were based on the guidelines of the Chinese Thyroid Association [16]. All surgeries were performed by 7 experienced thyroid surgeons at our center. Therapeutic central neck dissection (CND) was performed when preoperative ultrasound or intraoperative exploration suggests central lymph node metastasis. Prophylactic CND was performed when preoperative ultrasonography suggested negative lymph nodes. For patients who underwent hemithyroidectomy and were pathologically confirmed to have central lymph node metastasis, the routine practice at our center was close surveillance, with complementary total thyroidectomy performed for patients who experienced recurrence during postoperative follow-up. Lateral neck dissection was performed after a biopsy confirmed the presence of metastatic lateral cervical lymph nodes. TNM classification was determined using the 8th edition of the American Joint Committee on Cancer (AJCC) cancer staging system [17]. Post-total thyroidectomy radioactive iodine (RAI) remnant ablation was performed in patients at risk of recurrence, which is based on the postoperative ATA risk stratification system [18]. After the initial treatment, all patients were regularly followed up every three months for thyroid function tests and neck ultrasound in the first year postoperatively. Subsequently, follow-up visits will be adjusted to every six months or annually according to disease stability. If suspicious lesions are detected, follow-up frequency was increased.

The following clinical information was collected, including gender, age at diagnosis, tumor size, bilaterality, multifocality, capsule invasion, extrathyroidal extension, lymph node metastasis, distant metastasis, comorbidities, RAI treatment, TNM stage, recurrence, and recurrence-free survival (RFS) at the end of follow-up. The mode of initial presentation in patients (symptomatic presentation, incidental health examinations, or active screening due to family history or other reasons) was ascertained from medical records and telephone interviews. Bilaterality was defined as tumors present in both thyroid lobes. Multifocality was diagnosed when two or more tumors were found in one or both lobes. Any extension exceeding the thyroid gland was defined as extrathyroidal extension. Recurrence was diagnosed as newly detected structural lesions demonstrating high suspicion for malignancy on imaging and pathologically confirmed via ultrasound-guided biopsy or surgical resection. RFS was defined as the time from surgery to recurrence.

### Statistical analysis

Categorical variables were reported as frequencies and percentages. Normally distributed continuous variables were expressed as mean ± standard deviation (SD), while skewed distributed continuous variables were expressed as median and interquartile range (IQR). Chi-square test or Fisher’s exact test were used to evaluate categorical variables, while Student’s t test and Wilcoxon’s rank sum test were used to assess continuous variables. RFS was estimated using the Kaplan–Meier method, and the Log-rank test was used to analyze time-dependent variables. All statistical analyses were performed using Statistical Package for the Social Sciences software (version 26.0; SPSS, Inc., Chicago, IL, USA). A P value of < 0.05 was considered statistically significant.

## Results

### Patient characteristics

A total of 672 NMTC patients (224 familial and 448 sporadic patients) were included in our study. The proportion of females in FNMTC and SNMTC patients was 75.45% and 81.25%, respectively. The mean ages of patients with FNMTC and SNMTC were 45.76 ± 13.34 and 47.01 ± 11.28, respectively. In pathological classification, PTC accounted for the largest proportion, with 89.73% in the FNMTC group and 86.38% in the sporadic group. Other histopathologic types included FTC (7.59% vs. 10.72%) and OTC (2.68% vs. 2.90%). Total thyroidectomy was performed in 46.43% of patients with FNMTC and in 40.40% of patients with SNMTC. Therapeutic central neck lymph node dissections were performed in 87.05% of patients with FNMTC and 84.38% of patients with SNMTC. For the TNM stage, the FNMTC group and SNMTC group had similar T stage distributions, while the FNMTC group exhibited a significantly higher N stage (52.68% vs. 44.42%; *p* = 0.0432) than the SNMTC group. The media follow-up times were 70.85 months and 75.2 months in the FNMTC and SNMTC groups, respectively. In the FNMTC group, 20.98% were detected via active screening due to family history, while 13.84% presented with symptoms. In the SNMTC group, the corresponding proportions were 6.92% and 20.98%, respectively. RAI treatment was administered to 28.57% of patients in the FNMTC group and 22.10% in the SNMTC group. Table 1 presents the comparison of baseline clinical characteristics between FNMTC and SNMTC patients.

**Table 1:** Patient characteristics of FNMTC and SNMTC

Characteristics	FNMTC, n (%)224 (100)	SNMTC, n (%)448 (100)	*P*-value
Gender			0.0800
Female	169 (75.45)	364 (81.25)	
Male	55 (24.55)	84 (18.75)	
Age (years)	45.76 ± 13.34	47.01 ± 11.28	0.0964
Pathology			0.9002
PTC	203 (90.62)	401 (89.51)	
FTC	15 (6.70)	34 (7.59)	
OTC	6 (2.68)	13 (2.90)	
Extent of surgery			0.1362
Total thyroidectomy	104 (46.43)	181 (40.40)	
Hemithyroidectomy	120 (53.57)	267 (59.60)	
Lymph node dissection			0.8194
Central neck dissection	195 (87.05)	388 (86.61)	
Lateral neck dissection	23 (10.27)	43 (9.60)	
TNM Stage (AJCC 8th)			
T Stage			0.2101
T1+T2	193 (86.16)	369 (82.37)	
T3+T4	31 (13.84)	79 (17.63)	
N Stage			0.0432*
N0	106 (47.32)	249 (55.58)	
N1	118 (52.68)	199 (44.42)	
M Stage			0.3469
M0	215 (95.98)	436 (97.32)	
M1	9 (4.02)	12 (2.68)	
Mode of initial presentation			<0.0001*
Active screening	47 (20.98)	31 (6.92)	
Incidental health examination	146 (65.18)	323 (72.10)	
Symptomatic presentation	31 (13.84)	94 (20.98)	
Radioactive iodine			0.0650
Yes	64 (28.57)	99 (22.10)	
No	160 (71.43)	349 (77.90)	
Follow-up (months, media, IQR)	70.85 (43.53)	75.20 (49.25)	

*PTC* papillary thyroid carcinoma, *FTC* follicular thyroid carcinoma, *OCT* Oncocytic thyroid carcinoma, *FNMTC* familial non-medullary thyroid carcinoma, *SNMTC* sporadic non-medullary thyroid carcinoma

### Comparison between FNMTC and SNMTC groups

The comparison of clinicopathological characteristics and prognostic factors between FNMTC and SNMTC groups is presented in Table 2. Compared to the SNMTC group, the FNMTC group had a smaller tumor diameter (1.03 ± 0.60 vs. 1.15 ± 0.96 cm; *p* = 0.0255). In addition, the results showed that the FNMTC group had higher rates of multifocality (43.75% vs. 34.82%; *p* = 0.024), bilaterality (32.59% vs. 23.88%; *p* = 0.0163), and lymph node metastasis (52.68% vs. 44.42%; *p* = 0.0432) compared to the SNMTC group. No differences were found between the two groups regarding gender, age, tumor size ≤ 1 cm, lymph node metastasis, distant metastasis, capsular invasion, extrathyroidal extension, Hashimoto’s thyroiditis (HT), thyroid follicular nodular disease, and recurrence rate.

**Table 2:** Comparison of clinicopathological characteristics and prognostic factors between FNMTC and SNMTC patients

Characteristics	FNMTC, n (%)224 (100)	SNMTC, n (%)448 (100)	*P*-value
Age (years)			0.3529
<55 years	170 (75.89)	325 (72.54)	
≥55 years	54 (24.11)	123 (27.46)	
Tumor size			0.2025
≤1cm	157 (70.09)	292 (65.18)	
>1cm	67 (29.91)	156 (34.82)	
Tumor diameter	0.99 ± 0.54	1.15 ± 0.96	0.0255*
Multifocality			0.0240*
Yes	98 (43.75)	156 (34.82)	
No	126 (56.25)	292 (65.18)	
Bilaterality			0.0163*
Yes	73 (32.59)	107 (23.88)	
No	151 (67.41)	341 (76.12)	
Lymph node metastasis			0.0432*
Yes	118 (52.68)	199 (44.42)	
No	106 (47.32)	249 (55.58)	
Central lymph node metastasis			0.0212*
Yes	114 (50.89)	186 (41.52)	
No	110 (49.11)	262 (58.48)	
Lateral lymph node metastasis			0.7833
Yes	33 (14.73)	57 (12.72)	
No	191 (85.27)	391 (87.28)	
Distant metastasis			0.1565
Yes	9 (4.02)	12 (2.68)	
No	215 (95.98)	436 (97.32)	
Capsular invasion			0.6373
Yes	44 (19.64)	95 (21.21)	
No	180 (80.36)	353 (78.79)	
Extrathyroidal extension			0.4188
Yes	26 (11.61)	62 (13.84)	
No	198 (88.39)	386 (86.16)	
HT			0.9419
Yes	38 (16.96)	75 (16.74)	
No	186 (83.04)	373 (83.26)	
Thyroid follicular nodular disease			0.1008
Yes	114 (50.89)	198 (44.20)	
No	110 (49.11)	250 (55.80)	
Recurrence			0.5338
Yes	13 (5.80)	21 (4.69)	
No	211 (94.20)	427 (95.31)	

*FNMTC* familial non-medullary thyroid carcinoma, *SNMTC* sporadic non-medullary thyroid carcinoma, *HT* Hashimoto's thyroiditis

### Subgroup analysis according to affected family members

To determine whether the number of affected family members of FNMTC has an impact on disease aggressiveness, we stratified FNMTC patients into FNMTC-2 (160 patients) and FNMTC-3 (64 patients) subgroups and compared both groups to SNMTC patients (Table 3). There was no significant difference in clinicopathological features and prognostic factors between the FNMTC-2 and the SNMTC group. However, the FNMTC-3 group had a smaller tumor diameter (0.89 ± 0.46 vs. 1.15 ± 0.96 cm; *p* = 0.0350) and a younger age at diagnosis (43.76 ± 14.53 vs. 47.01 ± 11.28 years; *p* = 0.0390) compared to the SNMTC group. In addition, patients of the FNMTC-3 had higher rates of tumors ≤ 1 cm (78.13% vs. 65.18%; *p* = 0.0397), multifocality (50.00% vs. 34.82%; *p* = 0.0185), bilaterality (37.50% vs. 23.88%; *p* = 0.0195), lymph node metastasis (57.81% vs. 44.42%; *p* = 0.0444), and coexisting with thyroid follicular nodular disease (57.81% vs. 44.20%; *p* = 0.0409) than those observed in SNMTC patients. No significant difference was found in terms of age (< 55 years), distant metastasis, capsular invasion, extrathyroidal extension, HT, or recurrence rate.

**Table 3:** Comparison of clinicopathological characteristics and prognostic factors among SNMTC, FNMTC-2, and FNMTC-3 patients

Characteristics	SNMTC, n (%)448 (100)	FNMTC-2, n (%)160 (100)	*P*-value(F2-S)	FNMTC-3, n (%)64 (100)	*P*-value(F3-S)	*P*-value(F2-3)
Gender			0.0921		0.3744	0.8061
Female	364 (81.25)	120 (75.00)		49 (76.56)		
Male	84 (18.75)	40 (25.00)		15 (23.44)		
Age (years)			0.3619		0.6795	0.8434
<55 years	325 (72.54)	122 (76.25)		48 (75.00)		
≥55 years	123 (27.46)	38 (23.76)		16 (25.00)		
Age at diagnosis	47.01±11.28	46.02±12.69	0.3593	43.76±14.53	0.0390*	0.2500
Tumor size			0.6982		0.0397*	0.0967
≤1cm	292 (65.18)	107 (66.88)		50 (78.13)		
>1cm	156 (34.82)	53 (33.12)		14 (21.87)		
Tumor diameter	1.15 ± 0.96	1.08 ± 0.64	0.3762	0.89 ± 0.46	0.0350*	0.0343*
Multifocality			0.1471		0.0185*	0.2330
Yes	156 (34.82)	66 (41.25)		32 (50.00)		
No	292 (65.18)	94 (58.75)		32 (50.00)		
Bilaterality			0.0938		0.0195*	0.3213
Yes	107 (23.88)	49 (30.63)		24 (37.50)		
No	341 (76.12)	111 (69.37)		40 (62.50)		
Lymph node						
metastasis			0.1765		0.0444*	0.3304
Yes	199 (44.42)	81 (50.63)		37 (57.81)		
No	249 (55.58)	79 (49.38)		27 (42.19)		
Central lymph						
node metastasis			0.0853		0.0466*	0.4725
Yes	186 (41.52)	81 (50.63)		33 (51.56)		
No	262 (58.48)	79 (49.37)		31 (48.44)		
Lateral lymph						
node metastasis			0.6267		0.0211*	0.0201*
Yes	57 (12.72)	18 (11.25)		15 (23.44)		
No	391 (87.28)	142 (88.75)		49 (76.56)		
Distant metastasis			0.5561		0.0755	0.2801
Yes	12 (2.68)	5 (3.13)		4 (6.25)		
No	536 (97.32)	155 (96.87)		60 (93.75)		
Capsular invasion			0.4065		0.6842	0.3659
Yes	95 (21.21)	29 (18.13)		15 (23.44)		
No	353 (78.79)	131 (81.87)		49 (76.56)		
Extrathyroidal extension			0.2993		0.9615	0.4681
Yes	62 (13.84)	17 (10.63)		9 (14.06)		
No	386 (86.16)	143 (89.37)		55 (85.94)		
HT			0.8861		0.6889	0.6524
Yes	75 (16.74)	26 (16.25)		12 (18.75)		
No	373 (83.26)	134 (83.75)		52 (81.25)		
Thyroid follicular						
nodular disease			0.3914		0.0409*	0.1901
Yes	198 (44.20)	77 (48.13)		37 (57.81)		
No	250 (55.80)	83 (51.87)		27 (42.19)		
Recurrence			0.8714		0.1165	0.1482
Yes	21 (4.69)	7 (4.38)		6 (9.38)		
No	427 (95.31)	153 (95.62)		58 (90.62)		

*SNMTC* sporadic non-medullary thyroid carcinoma, *FNMTC* familial non-medullary thyroid carcinoma, *FNMTC-2* families with two affected members, *FNMTC-3* families with ≥3 affected members, *F2-S* SNMTC vs. FNMTC-2, *F3-S* SNMTC vs. FNMTC-3, *F2-F3* FNMTC-2 vs. FNMTC-3, *HT* Hashimoto's thyroiditis

Similarly, compared with the FNMTC-2 group, the FNMTC-3 group had a smaller tumor diameter (0.89 ± 0.46 vs. 1.08 ± 0.64 cm; *p* = 0.0343) but a higher rate of lateral lymph node metastasis (11.25% vs. 23.44%, *p* = 0.0201). However, no significant difference was observed between the FNMTC-2 and FNMTC-3 groups in other clinicopathological and prognostic factors (Table 3).

### Prognostic analysis for FNMTC and SNMTC groups

**Fig. 1:**
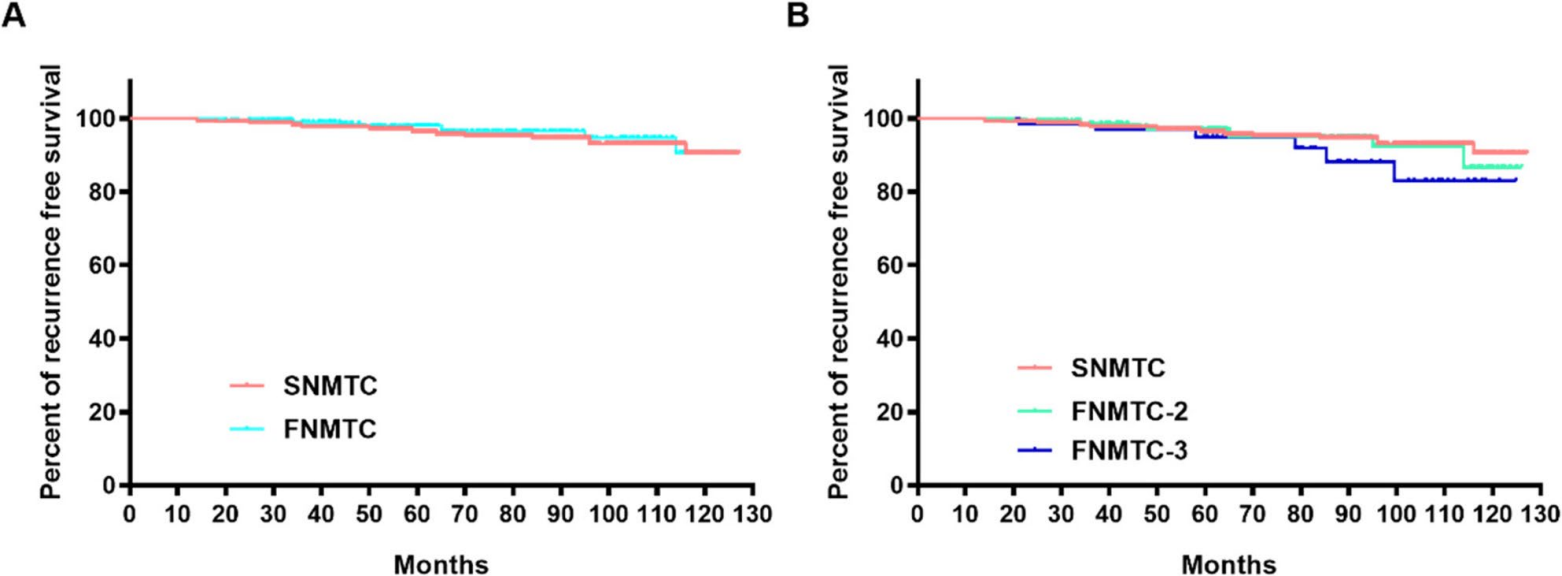
Recurrence-free survival curves of the SNMTC and FNMTC groups. **A** Comparison between FNMTC and SNMTC groups; **B** Comparison among SNMTC, FNMTC-2, and FNMTC-3 subgroups

For clinical outcomes, no patients in the FNMTC or SNMTC group died from the disease during the follow-up period. Disease recurrence occurred in 5.80% of FNMTC and 4.69% of SNMTC patients. RFS curves of the SNMTC and FNMTC groups are shown in Fig. 1. RFS was similar between the FNMTC and SNMTC groups (log-rank *p* = 0.4983; Fig. 1A), and no statistically significant differences in RFS were observed between SNMTC and the FNMTC-2 (*p* = 0.8406) or FNMTC-3 (*p* = 0.1770) subgroups, nor between FNMTC-2 and FNMTC-3 (*p* = 0.3414; Fig. 1B).

To investigate the potential influence of histological subtypes on clinicopathological characteristics and prognosis, we performed a subgroup analysis for PTC patients (Table S1). Within the PTC subgroup, the results of the subgroup analysis are largely consistent with those of the whole-cohort analysis, supporting our main conclusion.

## Discussion

Although FNMTC has been proposed as a potential distinct clinical entity [19], no clear consensus exists regarding its typical clinical behavior and outcomes. To the best of our limited knowledge, this study is one of the few large-scale single-center cohorts with long-term follow-up of FNMTC to date. Our findings show that FNMTC exhibits more aggressive clinical features than SNMTC overall, particularly when FNMTC is defined as FNMTC-3. In addition, our subgroup analysis stratified by the number of affected family members reveals that FNMTC-2 is phenotypically similar to SNMTC, whereas the FNMTC-3 subgroup exhibits more significant aggressive clinicopathological features. This result indicates the importance of the number of affected family members in the clinical assessment for FNMTC patients, providing a critical basis for risk stratification and personalized management for FNMTC.

Our study found that although FNMTC-2 exhibited more aggressive pathological features than SNMTC patients, most of these differences were not statistically significant. However, patients in the FNMTC-3 group were diagnosed at a younger age, along with significantly higher rates of tumors ≤ 1 cm, multifocality, bilaterality, lymph node metastasis, and coexisting follicular nodular disease compared to the SNMTC group. This difference might be explained by the view that true familial FNMTC-2 cases account for only part of its subgroup, with other sporadic cases diluting the overall aggressiveness [14]. In contrast, the FNMTC-3 subgroup was consistent with a higher probability of true hereditary cases, exhibiting distinct aggressive features compared to SNMTC [14, 20]. In addition, patients with a family history are more likely to undergo active screening (20.98% in FNMTC vs. 6.92% in SNMTC), leading to earlier diagnosis. This may contribute to the younger age and smaller tumor size in FNMTC-3. Therefore, the higher proportion of tumors ≤ 1 cm in FNMTC-3 is more likely attributed to earlier detection via family-based screening, rather than intrinsic tumor biology. In contrast, the higher rates of multifocality, bilaterality, and lymph node metastasis may reflect genuine biological aggressiveness. Furthermore, FNMTC-3 exhibits more aggressive pathological features but shows no difference in RFS compared to other subgroups. This paradox may be partly explained by the treatment received. Patients in the FNMTC-3 group, owing to their higher-risk features, were more likely to receive total thyroidectomy and RAI therapy in our cohort. These risk-based management strategies may have effectively mitigated their higher intrinsic recurrence risk, thereby influencing recurrence outcomes.

Our findings support the inclusion of the number of affected family members as a specific independent factor in the risk stratification of FNMTC. Multifocality of PTC has been proven as a risk factor for disease recurrence [21]. Kim’s study has shown that even unilateral multifocal PTC is significantly associated with a higher recurrence risk and poorer DFS [22]. In addition, lymph node metastasis was identified as a strong predictor of recurrence in NMTC patients [23]. Given the high rates of bilaterality, multifocality, and lymph node metastasis observed in the FNMTC-3 group, patients with FNMTC-3 should be classified as a high-risk subtype. While the 2025 ATA guidelines [24] recommend against routine prophylactic CND for most T1-stage, non-invasive, clinically node-negative cases. Given the high rates of central and lateral neck lymph node metastasis observed in FNMTC-3 patients, prophylactic central neck dissection may be considered in this subgroup [25], along with careful preoperative evaluation of lateral neck lymph nodes. Furthermore, FNMTC patients may require more intensive surveillance protocols [4, 24]. Patients with FNMTC-3 should also be informed about the potential risks for their first-degree relatives and the necessity of ultrasound screening. Ultrasound screening may also be considered for families with only two affected relatives but with other high-risk features.

Some studies have suggested that FNMTC should be defined as NMTC occurring in three or more first-degree relatives within a family [26, 27]. The main reason for this idea is that, since no definitive pathogenic genes for FNMTC have been identified [15], it’s impossible to rule out the possibility that some cases in FNMTC2 are sporadic. A series of studies have identified multiple gene variants within specific FNMTC families, including variants such as CHEK2 [28], FOXE1 [29], HABP2 [30], POT1 [31], and P21 [32]. Among them, the FOXE1 and the HABP2 genes have been validated by separate study groups [33]. However, these variants were reported only in a small number of FNMTC cases and have shown inconsistent associations [33, 34]. Consequently, increasing the number of affected family members required in defining the FNMTC has been used as a way to exclude sporadic clustering. According to the recent WHO definition [35], FNMTC is defined as patients from families with ≥ 3 first-degree relatives with follicular cell-derived thyroid carcinoma or ≥ 2 first-degree relatives with PTC combined with additional supportive features. These features include early onset (age < 33 years), affected relatives aged < 45 years, multifocality, bilaterality, young male patients, or combining with benign lesions. These additional features align with the clinicopathological features observed in the FNMTC-3 subgroup of this study, including a younger age of onset, a significantly higher prevalence of multifocality, bilaterality, and a combination with follicular nodular disease. This supports the rationale of the WHO classification for using ≥ 3 affected relatives as a reliable diagnostic criterion for true non‑syndromic FNMTC. Our findings suggest that the number of affected family members is associated with the aggressiveness of FNMTC. This is consistent with the WHO’s diagnostic approach for FNMTC, which combined the number of affected relatives and phenotypic characteristics, providing clinical empirical support for this diagnostic criterion.

The relationship between the number of affected family members and tumor aggressiveness remains controversial. Some studies suggested that FNMTC patients from families with three or more affected members exhibit more aggressive disease than those from families with two affected members, including smaller tumor diameters [36], higher risks of lymph node metastasis [37], and worse DFS [38]. A recent meta-analysis indicated that the risks of clinical aggressiveness and poor prognosis in FNMTC increased with the number of affected family members [13]. Some other studies reported that no significant difference was observed between affected family members and tumor aggressiveness in FNMTC [39, 40]. In our study, patients in the FNMTC-3 group were diagnosed at a younger age, along with significantly higher rates of tumors ≤ 1 cm, multifocality, bilaterality, lymph node metastasis, and coexisting follicular nodular disease compared to the SNMTC group. Additionally, the FNMTC-3 group had a higher rate of lateral lymph node metastasis compared to the FNMTC-2 group. This gradient of risk of lateral lymph node metastasis within FNMTC further indicates that disease aggressiveness may be closely associated with familial burden. Interestingly, despite the more aggressive clinicopathological features in the FNMTC-3 group, we did not observe a statistically significant difference in RFS between the FNMTC-3 and other groups. This finding can likely be attributed to several factors. FNMTC-3 patients, due to their more aggressive clinical features at diagnosis, may have received more intensive initial therapy or postoperative treatment compared to other patients [41]. These aggressive treatments might mitigate the expected difference in recurrence. In addition, the multifocality observed in FNMTC-3 might originate from synchronous development of multiple independent primary tumors with similar low aggressiveness, rather than intra-thyroidal spread from a single highly aggressive clone. Furthermore, the study defined disease recurrence as structural relapse. This definition was unable to identify disease recurrence evidenced by biochemical markers, resulting in an underestimation of the true recurrence rate. The low prevalence and limited follow-up duration of FNMTC-3 cases decreased the statistical power to detect differences in RFS.

This study has several limitations. Firstly, the study is retrospective, which may have limited the accuracy and completeness of the collected data. Although post-hoc power analysis indicated that the overall sample size met the statistical requirements for most primary endpoints, the limited sample size of FNMTC subgroups may have insufficient statistical power to detect differences in outcomes with small effect sizes. Secondly, metastatic and recurrent disease often progresses over longer periods. Thus, the lack of significant differences in distant metastasis and RFS may reflect delayed manifestation rather than true biological equivalence [13]. Furthermore, the treatment strategies, particularly the extent of surgery and use of RAI, were not standardized between the FNMTC and SNMTC groups. The higher rates of total thyroidectomy and RAI use in the FNMTC-3 group may have mitigated recurrence risk. These treatment differencesmay represent a potential confounding factor when comparing recurrence outcomes. Thirdly, PTC constitutes the majority among the pathological types in our cohort. Consequently, our findings primarily reflect the behavior of familial PTC. Future studies specifically addressing other subtypes (FTC and OTC) in the FNMTC are needed. Therefore, long-term follow-up of larger cohorts is necessary to compare survival outcomes in the future.

## Conclusion

This single-center retrospective study shows that FNMTC-3 patients exhibit unique clinicopathological features compared to SNMTC, while FNMTC-2 patients show no significant differences from SNMTC. FNMTC-3 patients exhibit a significantly younger age at diagnosis, smaller tumor diameter, and increased incidences of multifocality, bilaterality, lymph node metastasis, and coexisting follicular nodular disease. Although the study did not observe a significant difference in RFS among the groups in the follow-up period, the aggressive pathological features of FNMTC-3 warrant more vigilant surveillance. Larger, multi-center studies with long-term follow-up are essential to refine risk stratification and enable personalized treatment for FNMTC.

## Supplementary Information

Supplementary Material 1.

## Data Availability

The datasets used and/or analyzed during the current study are available from the corresponding author on reasonable request.

## Abbreviations

FNMTC: Familial non-medullary thyroid carcinoma
FNMTC-2: Families with 2 affected first-degree relatives having FNMTC
FNMTC-3: Families with ≥ 3 affected first-degree relatives having FNMTC
SNMTC: Sporadic non-medullary thyroid carcinoma
NMTC: Non-medullary thyroid carcinoma
PTC: Papillary thyroid carcinoma
ATC: Anaplastic thyroid carcinoma
FTC: Follicular thyroid carcinoma
OTC: Oncocytic thyroid carcinoma
US: Ultrasonography
FNA: Fine-needle aspiration
CND: Central neck dissection
AJCC: American Joint Committee on Cancer
SD: Standard deviation
IQR: Interquartile range
RFS: Recurrence-free survival
DFS: Disease-free survival
