# Supplementary material for: Clinical characteristics and prognosis of familial non-medullary thyroid carcinoma: a retrospective study of 98 families
Source: World J Surg Oncol. 2026 Apr 15;24:230. doi: 10.1186/s12957-026-04357-9 (PMC13196012; doi:10.1186/s12957-026-04357-9)
Supplement: Supplementary file 1 — Supplementary Material 1. [file 12957_2026_4357_MOESM1_ESM.pdf]

Table 1. Subgroup analysis for PTC patients..

Characteristics	SPTC, n (%) 401 (100)	FPTC, n (%) 203 (100)	P-value (F-S)	FPTC-2, n (%) 142 (100)	P-value (F2-S)	FPTC-3, n (%) 61 (100)	P-value (F3-S)	P-value (F2-3)
Gender			0.0978		0.1218		0.3503	0.8787
Female	329(82.04)	155(76.35)		108(76.06)		47(77.05)		
Male	72(17.96)	48(23.65)		34(23.94)		14(22.95)		
Age (years)			0.7984		0.8183		0.8779	0.9930
<55 years	306(76.31)	153(75.37)		107(75.35)		46(75.41)		
≥55 years	95(23.69)	50(24.63)		35(24.65)		15(24.59)		
Age at diagnosis	47.14 ± 11.15	45.16 ± 13.52	0.0550	45.77 ± 12.98	0.2263	43.57 ± 14.63	**0.0264***	0.2886
Tumor size			0.2468		0.6434		0.1010	0.2244
≤1cm	274(68.33)	148(72.91)		100(70.42)		48(78.69)		
>1cm	127(31.67)	55(27.09)		42(29.58)		13(21.31)		
Tumor diameter	1.12 ± 0.99	0.97 ± 0.54	**0.0432***	1.03 ± 0.58	0.2580	0.87 ± 0.45	**0.0495***	0.0644
Multifocality			**0.0218***		0.1137		**0.0280***	0.3480
Yes	145(36.16)	93(45.81)		62(43.66)		31(50.82)		
No	256(63.84)	110(54.19)		80(56.34)		30(49.18)		
Bilaterality			**0.0093***		**0.0450**		**0.0281***	0.5267
Yes	98(24.44)	70(34.48)		47(33.10)		23(37.70)		
No	303(75.56)	133(65.52)		95(66.90)		38(62.30)		
Lymph node metastasis			**0.0271***		0.0816		0.0836	0.6557
Yes	189(47.13)	115(56.65)		79(55.63)		36(59.02)		
No	212(52.87)	88(43.35)		63(44.37)		25(40.98)		
Central lymph node metastasis			**0.0121***		**0.0160***		0.2101	0.6770
Yes	176(43.89)	111(54.68)		79(55.63)		32(52.46)		
No	225(56.11)	92(45.32)		63(44.37)		29(47.54)		
Lateral lymph node metastasis			0.4032		0.7550		**0.0273***	**0.0349***
Yes	55(13.72)	33(16.26)		18(12.68)		15(24.59)		
No	346(86.28)	170(83.74)		124(87.32)		46(75.41)		
Distant metastasis			0.4257		0.5772		0.4112	0.6990
Yes	11(2.74)	8(3.94)		5(3.52)		3(4.92)		
No	390(97.26)	195(96.06)		137(96.48)		58(95.08)		
Capsular invasion			0.8297		0.6234		0.7214	0.5218

Yes	84(20.95)	41(20.20)		27(19.01)		14(22.95)		
No	317(79.05)	162(79.80)		115(80.99)		47(77.05)		
Extrathyroidal extension			0.5694		0.5016		0.9402	0.7086
Yes	54(13.46)	24(11.82)		16(11.26)		8(13.11)		
No	347(86.53)	179(88.18)		126(88.73)		53(86.89)		
Hashimoto's thyroiditis			0.9916		0.7828		0.6371	0.5479
Yes	69(17.21)	35(17.24)		23(16.20)		12(19.67)		
No	332(82.79)	168(82.76)		119(83.80)		49(80.33)		
Thyroid follicular nodular disease			0.1557		0.6006		**0.0473***	0.1475
Yes	182(45.39)	102(50.25)		67(47.18)		36(57.37)		
No	219(54.61)	101(49.75)		75(52.82)		25(42.62)		
Recurrence			0.6183		0.8913		0.2167	0.2518
Yes	18(4.49)	11(5.42)		6(4.23)		5(8.20)		
No	383(95.51)	192(94.58)		136(95.77)		56(91.80)		
RFS			0.5644		0.8069		0.2382	0.4344

SPTC: sporadic non-medullary thyroid carcinoma; FPTC: familial non-medullary thyroid carcinoma; FPTC-2: families with two affected members; FPTC-3: families with ≥3 affected members; F2-S: SPTC vs. FPTC-2; F3-S: SPTC vs. FPTC-3; F2-F3: FPTC-2 vs. FPTC-3;
